# Incidence and Predictors of Difficult Mask Ventilation in High-Risk Adult Population Scheduled for Elective Surgery: A Prospective Observational Study

**DOI:** 10.7759/cureus.22002

**Published:** 2022-02-07

**Authors:** Maha Khan, Ali S Siddiqui, Syed A Raza, Khalid Samad

**Affiliations:** 1 Anaesthesiology, Aga Khan University Hospital, Karachi, PAK; 2 Anaesthesiology, Aga Khan University, Karachi, PAK; 3 Anaesthesia and Critical Care, Aga Khan University Hospital, Karachi, PAK

**Keywords:** endotracheal intubation, mask, difficult intubation, difficult ventilation, airway

## Abstract

Introduction

Mask ventilation is one of the key components in the management of airway during general anaesthesia, particularly when laryngoscopy is challenging. Adequate mask ventilation provides anaesthesiologists a safe time in case of unanticipated or anticipated difficult airway situations. The aim of this study was to determine the incidence of difficult bag-mask ventilation and intubation in patients having three or more predictors for difficult mask ventilation (DMV) in adult patients scheduled for elective surgery under anaesthesia.

Methods

A total of 294 patients requiring endotracheal intubation for elective surgical procedure having three or more risk factors were evaluated for the presence of difficulty in bag-mask ventilation and intubation by the anaesthesiologist. Chi-square test or Fisher's exact test and a multivariable stepwise logistic regression model were performed to identify predictors of DMV. Crude and adjusted odds ratio with 95% confidence interval were reported.

Results

In this study, the average age of the patients was 53.59±13.32 years with a 2:1 male-to-female ratio. DMV and difficult intubation (DI) were observed in 31.6% and 3% of patients, respectively. Multivariate analysis identified history of snoring, BMI (>35 kg/m^2^), presence of beard and Mallampati III or IV as independent predictors for DMV. Patients with multiple factors (≥3 factors) had a threefold (OR=2.57) increased risk of difficulty in mask ventilation and a nearly fivefold (OR=4.63) increased risk of difficulty with intubation.

Conclusion

In our study, the incidence of DMV was observed in 93 (31.6%) patients and DI was found in 9 (3%) patients. A simple DMV risk score may help to predict DMV better, potentially improving safety during difficult airway management, decreasing morbidity and mortality associated with it.

## Introduction

Airway management is the primary responsibility of anaesthesiologist that includes mask ventilation, laryngoscopy, endotracheal intubation, and extubation. Failure to manage the airway appropriately may potentially be associated with increased morbidity and mortality resulting in hypoxic brain injury. In a recent literature review, difficult mask ventilation (DMV) incidence varies from 0.08% to 15% [1,2], whereas difficult laryngoscopy varies between 1.5% and 18% [3]. Despite its clinical importance, limited data are available from low-middle-income countries to predict DMV and intubation with accuracy. This prospective study was conducted to determine the incidence of DMV in patients having multiple risk factors for predicting DMV and its association with difficult intubation (DI).

## Materials and methods

Institutional Ethical Review Committee approval was taken before the conduct of the study (4101-Ane-ERC-16). We identified eight risk factors associated with a high incidence of difficulty in mask ventilation after reviewing the literature, which includes age more than 47 years, gender, BMI >35 kg/m^2^, history of obstructive sleep apnea (OSA), history of snoring, Mallampati III or IV, beard and limited jaw protrusion [2-5]. Patients having three or more risk factors were included in the study after taking the informed consent.

Patients who were edentulous, had a history of facial burn, trauma, or laceration, had a history of head and neck surgery, had a tracheostomy, required rapid sequence induction, were planning to use a supraglottic airway device for airway maintenance, planning to use fiberoptic intubation or planning to use video laryngoscope were all excluded.

The primary investigator assessed the oro-pharyngeal view and graded it according to the modified Mallampati test, in which patients were asked to open their mouth maximally and protrude the tongue without phonation when placed in a seated position [4]. The upper lip bite test was also performed to evaluate the limited jaw protrusion. The patient was kept in a sitting position and was asked to bite their upper lip with lower incisors and was graded [5].

All patients were pre-oxygenated with 100% oxygen for 3 minutes before induction and after application of American Society of Anesthesiologists (ASA) standard monitoring, including non-invasive blood pressure, electrocardiography and pulse oximetry. The induction agent and the use of a non-depolarizing muscle relaxant were as per the choice of the primary anaesthesiologist. Anaesthesiologists with less than two years of experience were not involved in the study. After induction of anaesthesia with propofol 2 mg/kg and atracurium 0.5 mg/kg, the patient was ventilated by bag mask using 40% oxygen and 60% nitrous oxides with isoflurane or sevoflurane until the patient was completely paralyzed for 3 minutes; 100% oxygen was used in patients with anticipated DMV. Difficulty in bag-mask ventilation was graded according to the operational definition and documented in the data collection form as follows: Grade I: easy to ventilate, Grade II: requiring nasopharyngeal or oral airway to ventilate, Grade III: difficult to ventilate or require two providers, Grade IV: unable to ventilate using all the above maneuvers. Grades III and IV are defined as DMV [6].

After complete relaxation and keeping the patient's head in a sniffing position, a laryngoscopy was performed. The laryngoscopic view was graded and noted by the primary anaesthesiologist as per the following operational definition: Grade I: glottis - full view; Grade II: glottis - exposed partly (anterior commissure not seen); Grade III: only epiglottis is seen; Grade IV: epiglottis not seen. Grades III and IV were documented as difficult laryngoscopy. The primary investigator collected all data regarding airway assessment and management till the airway was secured and the endotracheal tube placement was confirmed.

Sample size calculation was based on a previous study in which the frequency of patients with DMV who have three or more risk factors was observed to be 25.8% using PASS 2011 (version 11.04) [7]. Two hundred and ninety patients were needed to estimate the expected rate within a 5% margin of error with a 95% confidence interval (CI). Data were analyzed by Statistical packages for social science version 19 (IBM Corp, Armonk, NY). DMV and DI were the outcome variables. Quantitative point estimation was reported in terms of mean and standard deviation, i.e., age and BMI, while incidence and percentages were computed for qualitative point estimation and analyzed by chi-square test or Fisher's exact test. The crude odds ratio (OR) for each factor was reported by taking p ≤0.05 as significant. The multivariable stepwise logistic regression model was performed to identify predictors of DMV. Adjusted OR with 95% CIs were reported for significant predictors. Insignificant predictors were excluded from the model. The low rate of DI has not fulfilled the assumption of multivariable analysis, so only a univariate OR was computed for DI. A p-value of ≤0.05 was considered significant.

## Results

**Figure 1:**
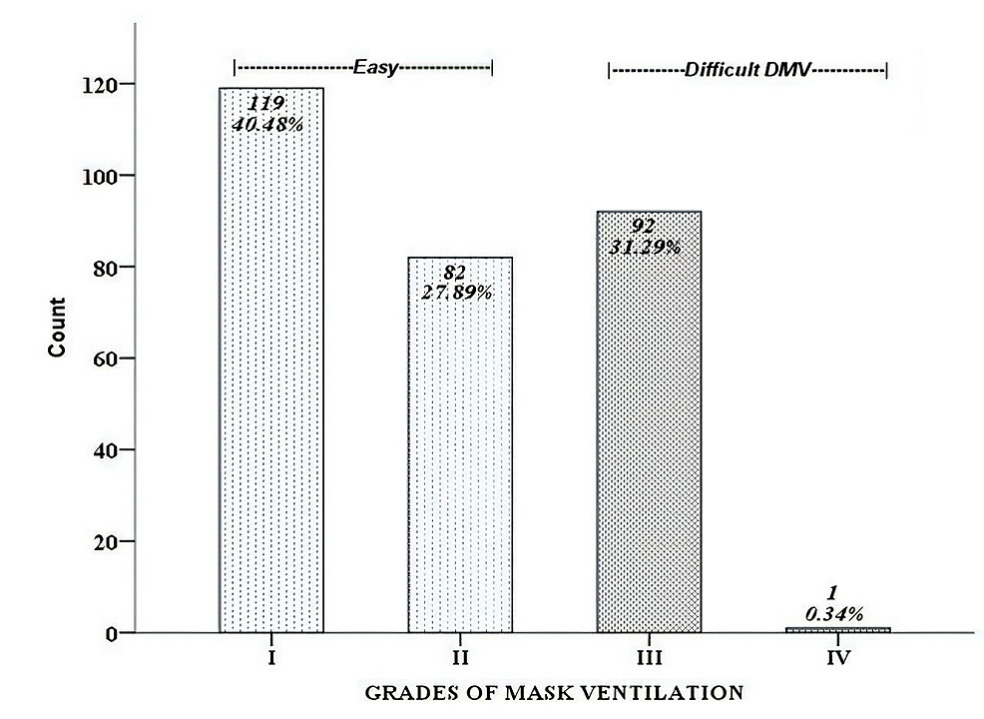
Grade of mask ventilation in patients having three or more predictors DMV, difficult mask ventilation.

A total of 294 patients were enrolled in the study; 200 (68%) were males and 94 (31.9%) were females. DMV was observed in 93 (31.6%) patients; 76 were male and 17 female, with Grade III mask ventilation in 92 (31.2%) patients and 1 (0.3%) patient had Grade IV mask ventilation (Figure 1). DI was observed in 9 (3%) patients; among them, five were male and four were female. All of them had Grade III intubation. In Table 1, crude OR showed that the male gender [OR=2.77; 95% CI: 1.53-5.04] and presence of beard [OR=2.19; 95% CI: 1.33-3.62] had significant difficulty in bag-mask ventilation, while other factors like age (>47 years), BMI (>35 kg/m^2^), history of OSA and snoring, Mallampati (III and IV) limited jaw protrusion were not statistically significant. None of the factors were statistically significant to justify DI because of the low rate of DI.

**Table 1:** Univariate analysis for predictors of DMV and DI *p-value < 0.01. NA, not applicable; BMI, body mass index; DMV, difficult mask ventilation; DI, difficult intubation; OR, odds ratio; CI, confidence interval.

Variables	N	DMV		DI	
		n (%)	OR [95% CI]	n (%)	OR [95% CI]
Age ≥47 (years)					
Yes	228	72 (31.6%)	0.98 [0.54-17.8]	7 (3.1%)	1.04 [0.21-5.0]
No	66	21 (31.8%)	Ref	2 (3%)	Ref
Gender					
Male	200	76 (38%)*	2.77 [1.53-5.04]	5 (2.5%)	0.57 [0.15-2.20]
Female	94	17 (18.1%)	Ref	4 (4.3%)	Ref
BMI ≥35 kg/m^2^					
Yes	114	38 (33.3%)	1.14 [0.68-1.89]	5 (4.4%)	2.01 [0.53-7.68]
No	180	55 (30.6%)	Ref	4 (2.2%)	Ref
History of obstructive sleep apnea					
Yes	7	1 (14.3%)	0.35 [0.042-2.97]	0 (0%)	NA
No	287	92 (32.1%)	Ref	9 (3.1%)	Ref
History of snoring					
Yes	258	86 (33.3%)	2.07 [0.87-4.92]	9 (3.5%)	NA
No	36	7 (19.4%)	Ref	0 (0%)	Ref
Mallampati III and IV					
Yes	234	77 (32.9%)	1.35 [0.72-2.54]	8 (3.4%)	2.09 [0.25-17]
No	60	16 (26.7%)	Ref	1 (1.7%)	Ref
Presence of beard					
Yes	138	56 (40.6%)*	2.19 [1.33-3.62]	3 (2.2%)	0.55 [0.136-2.26]
No	156	37 (23.7%)	Ref	6 (3.8%)	Ref
Limited jaw protrusion					
Yes	12	1 (8.3%)	0.188 [0.024-1.47]	1 (8.3%)	3.11 [0.35-27.12]
No	282	92 (32.6%)	Ref	8 (2.8%)	Ref

In multivariate analysis, the stepwise logistic regression model showed that history of snoring, BMI (>35 kg/m^2^), beard and Mallampati III or IV were strongly and significantly associated with DMV. The adjusted OR of these predictors indicated 2 to 3 times more chances of DMV (Table 2). Other factors like age (>47 years), gender, history of OSA and limited jaw protrusion were not significant, so they were excluded from the model.

**Table 2:** Factors associated with difficult mask ventilation in multivariable analysis Model accuracy=70.1%. Outcome variable was difficult mask ventilation. aOR, adjusted odds ratio; SE, standard error; CI, confidence interval.

Risk factors	Regression coefficient (SE)	p-Value	aOR [95%CI]
BMI >35 kg	0.81 (0.32)	0.012	2.26 [1.20-4.25]
History of snoring	1.113 (0.46)	0.016	3.05 [1.23-7.51]
Mallampati III or IV	0.68 (0.34)	0.045	1.99 [1.02-3.87]
Presence of beard	1.37(0.32)	0.0005	3.96 [2.09-7.47]

Table 3 exhibited that multiple risk factors (≥3) in patients were significantly associated with DMV and DI. The inflating trend of OR for four and five factors in patients had three to five times more predicted power of DMV. Patients who encountered both DMV and DI were four out of 294 (1.3%) and all of these cases also had three or more risk factors in which BMI (>35 kg/m^2^), history of snoring, Mallampati III or IV, presence of beard and age (>47 years) were observed.

**Table 3:** Association of number of risk factors involved in predicting DMV and DI *Significance. Data are presented as n (%). OR, odds ratio; CI, confidence interval; DMV, difficult mask ventilation; DI, difficult Intubation.

Three or more predictors	Total	DMV; n=93	p-Value	OR [95% CI]	DI; n=9	p-Value	OR [95% CI]
3	195	47 (24.1%)	-	Ref	3 (1.5%)	-	Ref
4	89	40 (44.9%)	0.0005*	2.57 [1.51-4.37]	6 (6.7%)	0.033*	4.63 [1.13-18.94]
5	10	6 (60%)	0.02	4.72 [1.27-17.45]	0 (0%)	NA	-

## Discussion

Unanticipated difficult airway remains one of the challenges faced by the anaesthesiologists in clinical practice. All currently available bedside airway screening tools for airway assessment have low sensitivities and high variability. Several airway assessment tools are available, but their reliability in predicting difficult airways varies in the literature [8,9]. In previous studies, greater focus was on the evaluation of predictors of difficult laryngoscopy and intubation. However, limited data are available on predictors of DMV.

In this study, the following observations were made:

1. The frequency of DMV was 31.6% (93 patients out of 294 patients), in the presence of three or more risk factors.

2. The frequency of DI was 3% (nine out of 294 patients) with Cormack Lehane Grade [III/IV] if three or more predictors were present preoperatively.

3. Greater difficulty in mask ventilation was observed in male patients having a beard with a p-value of 0.001 and 0.002, respectively.

4. None of the above risk factors were significant in predicting DI.

5. However, patients with four or more risk factors had 2.57 times difficulty in mask ventilation and about five times difficulty in intubation. Male gender and the presence of beard were found as independent risk factors, which were not correlated with the study of Langeron et al. [10] in which age >55 years, BMI >26 kg/m^2^, lack of teeth and snoring were also identified as independent risk factors. Kheterpal et al. [2] identified age greater than 57 years, BMI >30 kg/m^2^, Mallampati III/IV, presence of beard, sleep apnea, thick neck, snoring and limited jaw protrusion as independent risk factors of mask ventilation and intubation. This study confirms Langeron et al.'s [10] and Kheterpal et al.'s [2] observation that the presence of beard was an independent predictor of DMV.

The observed frequency of DMV in our study was markedly higher (31.6%) than that reported in the earlier studies (DMV ranges from 0.07% to 12.82%) [10,11]. This difference is most likely due to the lack of standardized definition used to grade mask ventilation and include only those having three or more risk factors of DMV. In all the studies mentioned above, predictors of DMV were identified via univariate analysis, difficulty in MV was assessed, and the predictors that were significant were then labeled as the independent risk factors for DMV.

Cattano et al. confirmed many factors that have been associated with difficult airway like age, short neck, facial hair, BMI, neck circumference and history of OSA. They suggested using a simple bedside predictive score to improve the accuracy of DMV prediction and patient safety [7]. Similarly, Prerana N Shah [12] in his study found snoring, atlanto-occipital extension Grade III, Mallampati Grade III or IV, Cormack and Lehane Grade III or IV and BMI >26 kg/m^2^ as independent predictors for DI but DI was found only in seven patients out 39 patients having DMV. In our study, multivariate analysis identified BMI >35 kg/m^2^, history of snoring, Mallampati III or IV and beard as independent risk factors for DMV, which shows that DI can be predicted if three or more risk factors are present. We are unable to identify a single risk factor that can predict DI. 

In the current study, DMV and DI situations together were observed in 1.3% of cases, which was the same as previously reported by Langeron et al. [10]. This classical situation in which the primary anaesthesiologist has difficulty in both mask ventilation and intubation is considered the most feared and challenging situation. The ability to predict such a situation would allow the anaesthesiologist to prepare for it with the alternative airway management techniques, such as laryngeal mask airway, fiberoptic and video laryngoscope. Kheterpal et al. [2] showed that risk increases by five times if the patient has four or more predictors as compared to three predictors for combined DMV and DI. Prakash et al. [13] observed the incidence of difficult laryngoscopy and intubation in 9.7% and 4.5%, respectively. Similar results were found in our study. More risk factors (≥4) (OR=4.63; 95%CI: 1.13-18.94) had more chance. Although Langeron et al. [10] have identified lack of teeth as a predictor for DMV, the reason for not considering the lack of teeth in our study as a risk factor for DMV was that in a larger trial done by Kheterphal et al. [2], they were unable to identify it as an independent predictor of DMV for both Grades III and IV.

Identifying predictors of DMV pre-operatively can help anaesthesiologists in improving the safety of airway management. Our study results may help to plan the technique of mask ventilation and intubation in patients with known predictors and can probably help reduce the morbidity and mortality related to difficult airways. Limited data are available in the Asian population for the incidence of difficulty in mask ventilation and intubation. None of the previous studies assessed patients already at risk for difficulty in mask ventilation and intubation. In this study, the sample size of patients with DMV and DI was less and the accuracy of such analysis was low, so more studies are required to predict these variables accurately. This was our study limitation. This study was single-centered, so multi-centered studies evaluating a large sample size are required to reach a definite conclusion. As this study involved the adult population, our results are not applicable to paediatric patients. There is a need for observational studies to identify predictors that can predict the difficulty in the pediatric population.

## Conclusions

Difficult airway management will remain a real challenge despite advancements in technology and the development of algorithms/guidelines. No single bedside test is available which can accurately predict difficult airway, especially in different settings and populations. The findings of our study demonstrate that DMV occurs in 31.6% of patients who have three or more risk factors, which might be a significant safety concern, particularly in challenging laryngoscopy and intubation settings. A simple bedside risk score will certainly help improve the prediction of DMV, improving patient safety during difficult airway management. Further prospective multicenter studies assessing a larger population who are at risk for difficulty in mask ventilation/intubation having three or more risk factors are needed to validate the results of previous studies and to make these observations as a part of airway management guidelines.

## References

[1] El-Orbany M, Woehlck HJ (2009). Difficult mask ventilation. Anesth Analg.

[2] Kheterpal S, Han R, Tremper KK, Shanks A, Tait AR, O'Reilly M, Ludwig TA (2006). Incidence and predictors of difficult and impossible mask ventilation. Anesthesiology.

[3] Farzi F, Abdolahzade M, Mirmansouri A, Nahvi H, Forghanparast K (2012). Difficult laryngoscopy; The predictive value of ratio of height to thyromental distance versus other common predictive tests of upper airway. Professional Med J.

[4] Cormack RS, Lehane J (1984). Difficult tracheal intubation in obstetrics. Anaesthesia.

[5] Khan ZH, Kashfi A, Ebrahimkhani E (2003). A comparison of the upper lip bite test (a simple new technique) with modified Mallampati classification in predicting difficulty in endotracheal intubation: a prospective blinded study. Anesth Analg.

[6] Han R, Tremper KK, Kheterpal S, O'Reilly M (2004). Grading scale for mask ventilation. Anesthesiology.

[7] Cattano D, Killoran PV, Cai C, Katsiampoura AD, Corso RM, Hagberg CA (2014). Difficult mask ventilation in general surgical population: observation of risk factors and predictors. F1000Res.

[8] Roth D, Pace NL, Lee A, Hovhannisyan K, Warenits AM, Arrich J, Herkner H (2018). Airway physical examination tests for detection of difficult airway management in apparently normal adult patients. Cochrane Database Syst Rev.

[9] Roth D, Pace NL, Lee A, Hovhannisyan K, Warenits AM, Arrich J, Herkner H (2019). Bedside tests for predicting difficult airways: an abridged Cochrane diagnostic test accuracy systematic review. Anaesthesia.

[10] Langeron O, Masso E, Huraux C, Guggiari M, Bianchi A, Coriat P, Riou B (2000). Prediction of difficult mask ventilation. Anesthesiology.

[11] el-Ganzouri AR, McCarthy RJ, Tuman KJ, Tanck EN, Ivankovich AD (1996). Preoperative airway assessment: predictive value of a multivariate risk index. Anesth Analg.

[12] Shah PN, Sundaram V (2012). Incidence and predictors of difficult mask ventilation and intubation. J Anaesthesiol Clin Pharmacol.

[13] Prakash S, Kumar A, Bhandari S, Mullick P, Singh R, Gogia AR (2013). Difficult laryngoscopy and intubation in the Indian population: an assessment of anatomical and clinical risk factors. Indian J Anaesth.

